# Chemical Composition, Repellent, and Oviposition Deterrent Potential of Wild Plant Essential Oils against Three Mosquito Species

**DOI:** 10.3390/molecules29112657

**Published:** 2024-06-04

**Authors:** Muhammad Ghazanfar Abbas, Muhammad Azeem, Muhammad Umar Bashir, Fawad Ali, Raimondas Mozūratis, Muhammad Binyameen

**Affiliations:** 1 Laboratory of Insect Chemical Ecology, Department of Entomology, Faculty of Agricultural Sciences and Technology, Bahauddin Zakariya University, Multan 60800, Pakistan; ghazanfarentomologist@gmail.com (M.G.A.); mumarbashir31@gmail.com (M.U.B.); 2 Department of Chemistry, COMSATS University Islamabad, Abbottabad Campus, Abbottabad 22060, Pakistan; muhazeem@cuiatd.edu.pk (M.A.); fawad36fy@gmail.com (F.A.); 3 Department of Zoology, Stockholm University, SE-10691 Stockholm, Sweden; 4 Laboratory of Chemical and Behavioral Ecology, Institute of Ecology, Nature Research Centre, LT-08412 Vilnius, Lithuania

**Keywords:** *Lantana camara*, eco-friendly, mosquito biting deterrent, *Aedes aegypti*, *Anopheles gambiae*, *Culex quinquefasciatus*

## Abstract

In this study, the chemical composition, repellent, and oviposition deterrent effects of five plant essential oils (EOs) extracted from *Lantana camara* (Verbenaceae), *Schinus terebinthifolia* (Anacardiaceae), *Callistemon viminalis* (Myrtaceae), *Helichrysum odoratissimum* (Asteraceae), and *Hyptis suaveolens* (Lamiaceae) were evaluated against *Aedes aegypti*, *Anopheles gambiae*, and *Culex quinquefasciatus*. When tested at 33.3 µg/cm^2^, *L. camara*, *S. terebinthifolia*, *C. viminalis*, and *H. odoratissimum* were effective repellents against *Ae. aegypti* (89%, 91%, 90%, and 51% repellency, respectively), but they were less repellent against *An. gambiae* (66%, 86%, 59%, and 49% repellency, respectively). Interestingly, *L. camara*, *S. terebinthifolia*, *C. viminalis*, and *H. odoratissimum* exhibited 100% repellency against *Cx. quinquefasciatus* at 33.3 μg/cm^2^. In time-span bioassays performed at 333 μg/cm^2^, the EO of *L. camara* exhibited 100% repellence against *Ae. aegypti* and *An. gambiae* for up to 15 min and against *Cx. quinquefasciatus* for 75 min. The oviposition bioassays revealed that *L. camara* exhibited the highest activity, showing 85%, 59%, and 89% oviposition deterrence against *Ae. aegypti*, *An. gambiae*, and *Cx. quinquefasciatus*, respectively. The major compounds of *L. camara*, *S. terebinthifolia*, and *C. viminalis* were *trans*-β-caryophyllene (16.7%), α-pinene (15.5%), and 1,8-cineole (38.1%), respectively. In conclusion, the *L. camara* and *S. terebinthifolia* EOs have the potential to be natural mosquito repellents.

## 1. Introduction

Mosquitoes are a significant public health concern because they can serve as vectors of dengue fever, filariasis, Japanese encephalitis, chikungunya, malaria, and yellow fever [1]. The *Aedes* mosquitos are known for their painful and persistent bites and can transmit pathogens causing dengue, chikungunya, Zika, and yellow fever diseases [2]. The *Anopheles* mosquitos are considered the primary vectors of diseases such as filariasis and malaria, with an estimated 300–500 million malaria cases yearly [3]. The *Culex* mosquitoes are the vectors of harmful diseases affecting birds and humans. These can serve as vectors of lymphatic filariasis, Saint Louis encephalitis, West Nile virus in humans, and avian malaria in birds [4].

Synthetic chemicals, while commonly used to control vector mosquitoes, have shown a decrease in efficacy against many species of mosquitoes due to their long-term use. Moreover, they pose risks to mammals, human health, and the environment [5,6]. In addition to chemical insecticides, mosquito repellents are also used to prevent mosquito bites. Since the late 1950s, DEET (*N*, *N*-diethyl-meta-toluamide) has been widely used as a common mosquito repellent [7], and some adverse effects, including skin irritation, encephalopathy in children, and allergic reactions have been reported [8,9]. Considering the harmful effects of synthetic insecticides and repellents, there is a need to develop phytochemical-based products to control these vectors of deadly diseases.

Plant-based products are considered safe to use and have either no harmful effects or low harmful effects on humans and the environment [10]. Several plant-based materials have proven extremely effective against blood-sucking insects, showing good repellent and insecticidal potential against mosquitoes [11,12]. Previously, several studies demonstrated the effectiveness of plant-derived essential oils (EOs) against mosquitoes (Table S1). Java citronella oil has been registered by the United States Environmental Protection Agency as an insect repellent due to its efficacy and low toxicity [13]. Another example is a repellent formulation derived from the leaves of the Australian lemon-scented gum tree *Corymbia citriodora*, in which one of the active ingredients is a monoterpene *p*-menthane-3,8-diol (PMD). In April 2005, the U.S. Centers for Disease Control and Prevention endorsed two non-DEET mosquito repellents, including PMD [14]. The estimated market value for plant-based chemicals is $700 million, or 45,000 tons, of the global pesticide production. Furthermore, these can be used without harming people or the environment because of their biodegradable nature [15]. Given the activity of plant EOs against harmful insects, the current study aimed to examine the repellent and oviposition deterrent potential of EOs from five plant species, including *Lantana camara* L. (Lamiales: Verbenaceae), *Schinus terebinthifolia* Raddi (Sapindales: Anacardiaceae), *Callistemon viminalis* Don. (Myrtales: Myrtaceae), *Helichrysum odoratissimum* (L.) (Asterales: Asteraceae), and *Hyptis suaveolens* (L.) (Lamiales: Lamiaceae), against adult female mosquitoes of three species (Diptera: Culicidae): *Aedes aegypti* (L.), *Anopheles gambiae* s. l. Giles, and *Culex quinquefasciatus* Say.

## 2. Results

### 2.1. Yield (%) of EOs

The aerial parts of *H. odoratissimum* yielded the highest amount of EO, while the *S. terebinthifolia* leaves consisted of the least amount (Table 1).

### 2.2. Repellency Results

**Figure 1:**
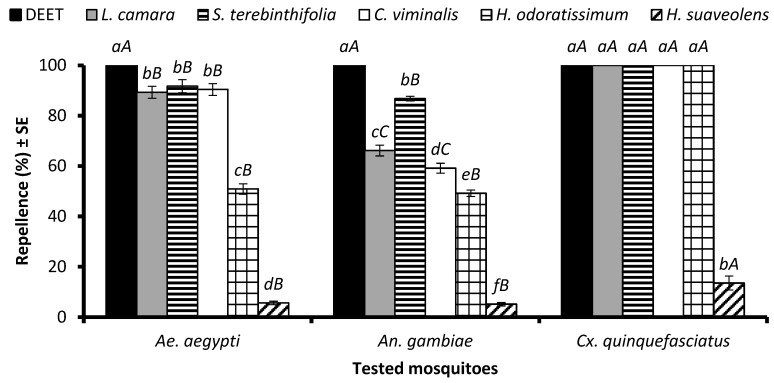
Repellency of positive control DEET and EOs at 33.3 µg/cm^2^ against *Aedes aegypti*, *Anopheles gambiae* s. l., and *Culex quinquefasciatus* mosquito females. Lowercase letters above the columns represent significant differences (*p* < 0.05) among tested EOs against particular mosquito species, while uppercase letters represent the significant difference (*p* < 0.05) among mosquitoes towards a particular EO, according to the ANOVA post-hoc Tukey test for each mosquito species and each EO separately. Error bars represent the standard error (*n* = 5).

An ANOVA revealed that the EO type significantly affected repellency against *Ae. aegypti* (df = 7, F = 586, *p* < 0.001), *An. gambiae* s. l. (df = 7, F = 973, *p* < 0.001), and *Cx. quinquefasciatus* (df = 7, F = 1634, *p* < 0.001) females when tested at a dose of 33.3 µg/cm^2^. DEET showed 100% repellence towards mosquitoes of all three species. The EOs of *L. camara*, *S. terebinthifolia*, and *C. viminalis* showed similar repellency (*p* > 0.05) of around 90% and were the most efficient after DEET, followed by the EO of *H. odoratissimum*, which displayed 52% repellent activity (Figure 1). The EO of *H. suaveolens* revealed significantly lower repellency (5.6%) against *Ae. aegypti* females. The EO of *S. terebinthifolia* was the second most repellent formulation against *An. gambiae* after DEET. The EOs of *L. camara*, *S. terebinthifolia*, *C. viminalis*, *H. odoratissimum*, and the positive control DEET showed 100% repellency against *Cx. quinquefasciatus*, while the EO distilled from *H. suaveolens* was the least efficient against all the tested mosquito species (Figure 1). Overall, the strongest repellent response was observed for *Cx. quinquefasciatus* compared to *Ae. aegypti* and *An. gambiae* females.

In the screening bioassay with a 1% *w*/*v* solution (33.3 µg/cm^2^ tested dose), the EOs of *L. camara*, *S. terebinthifolia*, *C. viminalis*, and *H. odoratissimum* showed more than 50% repellency against all mosquito species and were further investigated to determine the maximum period of repellent activity.

#### 2.2.1. Time-Span Repellency against *Ae. aegypti* Mosquito Females

**Figure 2:**
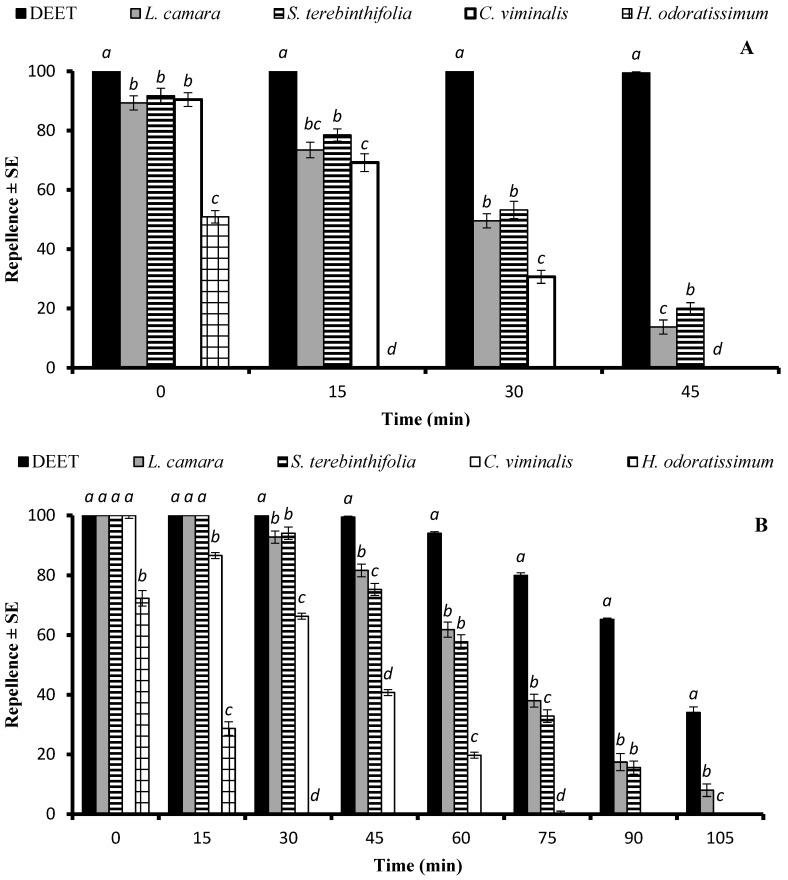
Repellent longevity of DEET and four EOs against *Aedes aegypti* females at the tested doses of 33.3 µg/cm^2^ (**A**) and 333 µg/cm^2^ (**B**). Different letters on bars indicate significant differences (*p* < 0.05) between the repellency of different substances tested after specified time intervals independently, according to the ANOVA post-hoc Tukey test. “SE” stands for standard error (*n* = 5).

The statistical analysis of the data revealed a significant impact of the type of EO on repellency against *Ae. aegypti* at doses of 33.3 µg/cm^2^ (df = 4, F = 138, *p* < 0.001), and 333 µg/cm^2^ (df = 4, F = 187, *p* < 0.001). DEET was the most efficient repellent at all tested time points. The EOs of *L. camara* and *S. terebinthifolia* were active over 45 min, displaying an extended repellent activity compared to the EO of *C. viminalis*, which was active for over 30 min. The EO of *H. odoratissimum* showed 50% repellency, which registered for 15 min (Figure 2A). At the dose of 333 µg/cm^2^, DEET, as well as the EOs of *L. camara*, *S. terebinthifolia*, and *C. viminalis*, showed statistically similar repellency (*p* > 0.05) when tested immediately after application (Figure 2B). The EOs of *L. camara* and *S. terebinthifolia* remained repellent above the 50% level for over 60 min and exhibited over 10% activity for 105 min and 90 min, respectively, while the repellent activity of the positive control DEET remained at 34% after 105 min. The shortest repellency period was observed for the EO of *H. odoratissimum* (Figure 2B).

#### 2.2.2. Time-Span Repellency against *An. gambiae* s. l. Mosquito Females

**Figure 3:**
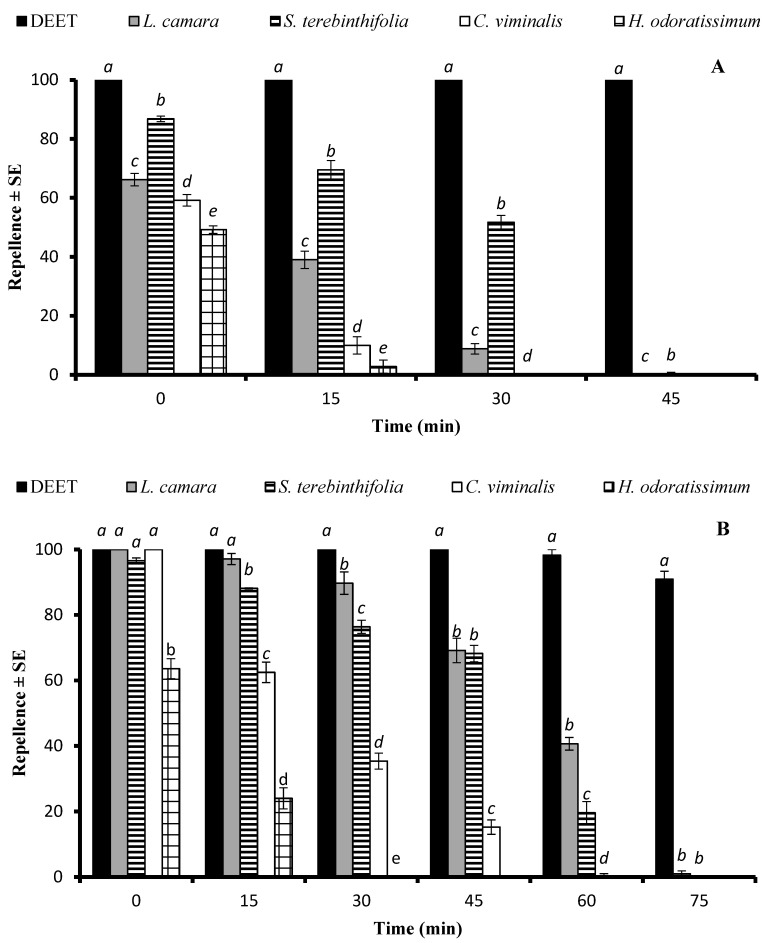
Repellent longevity of DEET and four EOs against *Anopheles gambiae* s. l. females at the tested doses of 33.3 µg/cm^2^ (**A**) and 333 µg/cm^2^ (**B**). Different letters on bars indicate significant difference (*p* < 0.05) between the repellency of different samples tested after specified time intervals independently, according to the ANOVA post-hoc Tukey test. “SE” stands for standard error (*n* = 5).

An ANOVA revealed that the EO type significantly affected repellency at doses of 33.3 µg/cm^2^ (df = 4, F = 331, *p* < 0.001), and 333 µg/cm^2^ (df = 4, F = 210, *p* < 0.001). The EO of *S. terebinthifolia* showed over 50% repellent activity, while the efficiency of the second most repellent EO, distilled from the *L. camara* plants, decreased from 66.2% to 8.8% 30 min after post-treatment (Figure 3A). The EOs of *C. viminalis* and *H. odoratissimum* showed the shortest repellent effect and were active for only up to 15 min. The positive control DEET displayed 100% repellency, which lasted for over 45 min.

At the higher dose of 333 µg/cm^2^, the EOs of *L. camara*, *S. terebinthifolia*, and *C. viminalis* showed statistically similar repellence (*p* > 0.05) just after the treatment compared to that of DEET, while after a 15 min period, the efficiency of the *C. viminalis* and *H. odoratissimum* EOs dropped significantly (*p* < 0.05) compared to those of DEET and *L. camara*. The EOs of *L. camara* and *S. terebinthifolia* exhibited an active time span of 60 min, whereas the repellent effect of the *C. viminalis* EO lasted for 45 min (Figure 3B).

#### 2.2.3. Time-Span Repellency against *Cx. quinquefasciatus* Mosquito Females

**Figure 4:**
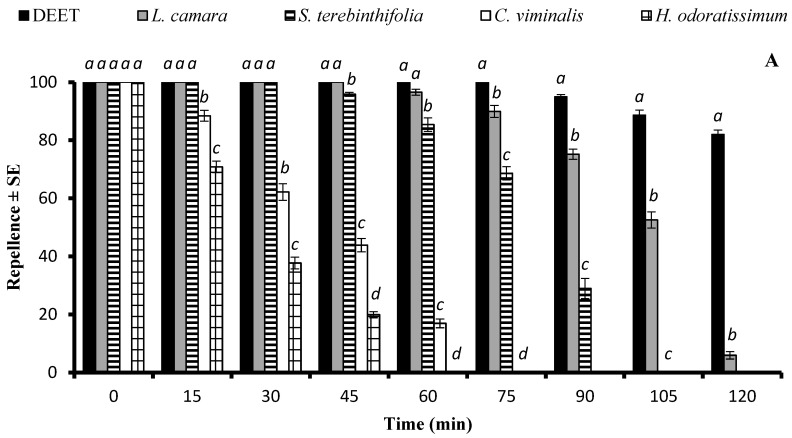

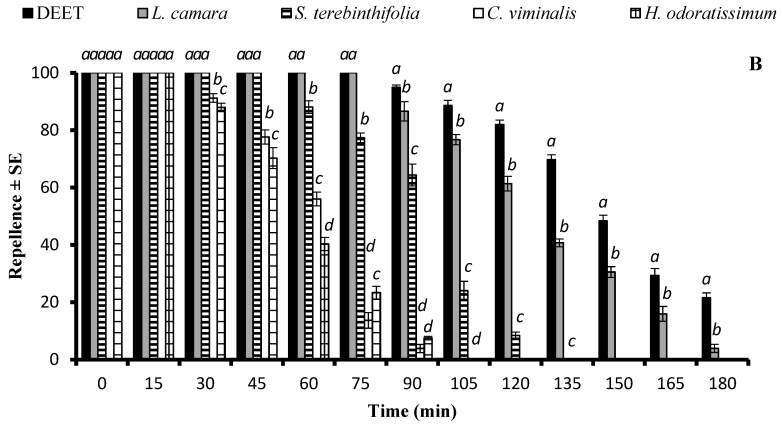
Repellent longevity of DEET and four EOs against *Culex quinquefasciatus* females at the tested doses of 33.3 µg/cm^2^ (**A**) and 333 µg/cm^2^ (**B**). Different letters on bars indicate significant differences (*p* < 0.05) between the repellency of different substances tested after specified time intervals independently, according to the ANOVA post-hoc Tukey test. “SE” stands for standard error (*n* = 5).

There was a significant difference in the repellent effects of essential oils against *Cx. quinquefasciatus* at the doses of 33.3 µg/cm^2^ and 333 µg/cm^2^. At 33.3 µg/cm^2^, *L. camara*, *S. terebinthifolia*, *C. viminalis*, and *H. odoratissimum* EOs showed a repellent effect similar to that of DEET (*p* > 0.05) immediately after application. The EOs of *L. camara* and *S. terebinthifolia* exhibited long-lasting repellent effects until 120 min and 90 min, respectively, and statistically similar repellent efficiency (*p* > 0.05) to that of DEET for 45 min and 30 min, respectively. The EOs of *C. viminalis* and *H. odoratissimum* had a lower repellent effect that lasted up to 60 min and 45 min, respectively (Figure 4A). The application of EOs at the higher dose of 333 µg/cm^2^ extended the active time span of *L. camara*, *S. terebinthifolia*, *C. viminalis*, and *H. odoratissimum* for up to 180 min, 120 min, 90 min, and 90 min, respectively. The positive control DEET and the EO of *L. camara* showed 100% repellency for 75 min, after which the efficiency of both substances started to decrease at different rates, resulting in a significantly higher repellency of DEET (Figure 4B).

### 2.3. Oviposition Deterrence of EOs

**Figure 5:**
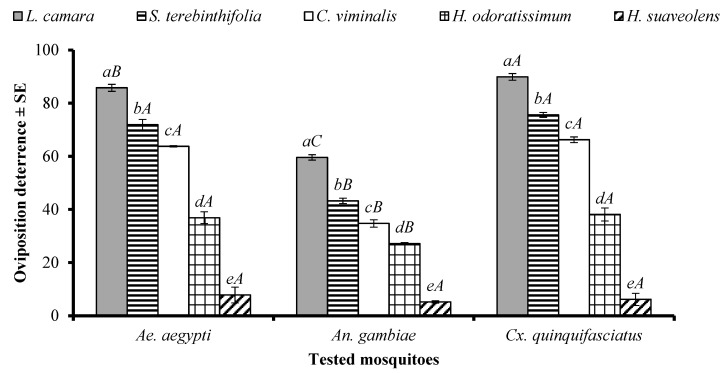
Oviposition behaviour-modifying effect of five EOs at the tested concentration of 300 µg/cm^2^ against *Aedes aegypti*, *Anopheles gambiae* s. l., and *Culex quinquefasciatus* mosquitos. Different lowercase letters above the columns represent significant differences (*p* < 0.05) between the tested EOs against particular mosquito species independently. Different uppercase letters on the bars represent significant differences (*p* < 0.05) between the oviposition behaviour of different mosquito species in the presence of a particular EO independently. An ANOVA post-hoc Tukey test was employed for each type of comparison separately. Error bars represent the standard error (*n* =5).

An ANOVA revealed that the EO type significantly affected the oviposition of *Ae. aegypti* (df = 4, F = 360, *p* < 0.001), *An. gambiae* s. l. (df = 4, F = 348, *p* < 0.001), and *Cx. quinquefasciatus* (df = 4, F = 968, *p* = 0) females. The EOs of *L. camara*, *S. terebinthifolia*, *C. viminalis*, and *H. odoratissimum* showed the same pattern of oviposition deterrence towards the females of *Ae. aegypti*, *An. gambiae* s. l., and *Cx. quinquefasciatus*, and the efficiency of these substances differed significantly from each other (Figure 5). The *L. camara* EO showed a higher oviposition deterrence against *Cx. quinquefasciatus*, followed by *Ae. aegypti* and *An. gambiae*. In the case of the *S. terebinthifolia*, *C. viminalis*, and *H. odoratissimum* EOs, a higher oviposition deterrence was observed against *Ae. aegypti*, and *Cx. quinquefasciatus*, as compared to *An. gambiae* (Figure 5). The EO of *H. suaveolens* showed the least oviposition deterrence towards the tested species of mosquitoes (Figure 5).

### 2.4. Chemical Profile of EOs

The most abundant compounds in the *L. camara* EO were *trans*-β-caryophyllene (16.7%), sabinene (16.5%), 1,8-cineole (13.1%), α-humulene (8.6%), and nerolidol (5.5%) (Table 2). The *S. terebinthifolia* EO comprised 15.5% α-pinene, 14% limonene, 12.4% α-phellandrene, 11.5% *p*-cymene, and 8.4% spathulenol. The *C. viminalis* EO was dominated by 1,8-cineole (38.1%) and α-pinene (34.2%), followed by *p*-cymene (9%) and α-terpineol (4%) (Table 2).

## 3. Discussion

Plant-based products can be used as repellents against mosquitoes. Repellent formulations derived from EOs have gained the attention of consumers and are considered safe to use compared to synthetic repellents. In the present study, five aromatic plant EOs were investigated for their mosquito repellent and oviposition deterrent activities against the females of three mosquito species. Overall, the host-seeking and oviposition behaviour of *Cx. quinquefasciatus* females was highly affected when exposed to the different EOs compared to *Ae. aegypti* and *An. gambiae* s. l. females, with *An. gambiae* s. l. showing the most resistance towards the tested EOs. The difference in the behavioural response of different mosquito species towards the same substance could be explained based on the presence of mismatched types of chemoreceptors in mosquito species. The findings of the current study are consistent with previously published reports, where mosquito species behaved differently when tested against the same substances [16,17]. Similar results were also found when three different stored-grain pest insects were exposed to *Artemisia sieberi* essential oil [18]. Previously, Amer and Mehlhorn [16] observed that the *Cx. quinquefasciatus* mosquitoes were found to be more sensitive to EOs compared to *Ae. aegypti*. Kweka, et al. [19] also demonstrated that the arms of volunteers treated with propylene glycol carbonate and DEET had a higher protective efficacy against *Cx. quinquefasciatus* compared to *An. gambiae*. A previous study demonstrated that the differential behavioural responses of different mosquito species towards the same test substances might be due to distinct sensitivities of their chemoreceptors [16].

Among all tested EOs, *L. camara* EO showed the highest repellency against all three mosquito species for an extended period of time. The effective and prolonged repellent activity of this EO could be attributed to the additive effect of β-caryophyllene, sabinene, 1,8-cineole, α-humulene, and nerolidol. However, the synergistic effects of the above-mentioned compounds with the minor compounds could not be ruled out. Similarly, a previous study reported that β-caryophyllene and nerolidol showed some repellency towards female *Ae. aegypti* when tested individually, whereas the combination of β-caryophyllene and nerolidol exhibited an excellent mosquito-repellent activity for an extended period of time [20]. To our knowledge, very few studies have described the mosquito-repellent activity of the *L. camara* EO or extracts. For example, *L. camara* flowers extracted in coconut oil exhibited 94% repellence against *Ae. aegypti* and *Ae. albopictus*; however, the concentration of the test substance was not given [21]. Keziah, et al. [22] reported that the hexane, ethyl acetate, and methanolic extracts of *L. camara* leaves exhibited 100% repellency towards *Ae. aegypti* females for 60 min when tested at 8000 µg/cm^2^. In another study, the solvent extracts and EO of *L. camara* showed repellent activity against *Ae. aegypti*, lasting for 225 min at a dose of 1660 µg/cm^2^ [23]. In the current study, the *L. camara* EO exhibited 100% protection for 15 min and 75 min against *Ae. aegypti* and *Cx. quinquefasciatus*, respectively, when tested at 333 µg/cm^2^. Here, we used a concentration five times lower compared to that of Bhargava et al. [23] and 24 times lower than that of Keziah et al. [22] but still, the bioactivity was comparable to the previous results. The difference in the bioactivity of the plant species could be explained based on the extraction method, the specific chemistry of the extracts due to the chemical variation within species, and the concentration of the tested products. Many studies have shown that the concentration of a substance plays a significant role in determining its bioactivity [24,25,26,27]. 

The EO of *S. terebinthifolia* provided complete protection against *Ae. aegypti* and *Cx. quinquefasciatus* for 15 and 45 min, respectively; however, it did not show complete protection against *An. gambiae* at any tested concentration. The observed repellent activity of *S. terebinthifolia* EO could be associated with the synergistic effect of diverse types of compounds, including α-pinene, limonene, α-phellandrene, *p*-cymene, spathulenol, elixene, and β-elemene. There is no previous study reporting the mosquito repellent activity of the *S. terebenthifolia* leaves EO. However, a previous study from Saudi Arabia demonstrated that the EO extracted from *S. terebinthifolia* fruits provided complete protection against *Cx. pipiens* for 120 min at a concentration of 0.50 μL/cm^2^ (~500 µg/cm^2^), whereas the nanoemulsion made from the same EO provided complete protection against *Cx. pipiens* for 150 min [28]. The major compounds in the *S. terebinthifolia* fruit EO were 31.2% 3-carene, 15.3% α-pinene, 11.2% α-phellandrene, 9.3% limonene, and 3.7% α-terpineol [28]. This composition was quite different from the chemical composition of the EO reported in the current study. The difference in the efficacy of *S. terebinthifolia* EOs reported in the current study and previously could be explained based on their different chemistry, probably due to the EOs being extracted from different parts of the plant, as well as the plants being grown under different conditions [29]. In addition, mosquito species respond differently to the same substance, which might explain the difference in bioactivity of *S. terebinthifolia* against the mosquito species tested in the current and previous studies. The fact that a substance can have different biological effects against different organisms is well documented [18]. 

The EO of *C. viminalis* showed the third-best repellent activity after *L. camara* and *S. terebinthifolia* against the *Ae. aegypti*, *An. gambiae*, and *Cx. quinquefasciatus*. The repellent activity of the *C. viminalis* EO was found to be dose-dependent and exhibited the best activity against all mosquito species at higher concentrations. In addition, its repellency against *Cx. quinquefasciatus* was long-lasting compared to the other two mosquito species. The most abundant compounds in the EO of *C. viminalis* were 1,8-cineole, α-pinene, *p*-cymene, and α-terpineol, which could be the reason for the good, but short-term repellent activity of *C. viminalis*. 1,8-cineole, α-pinene, and *p*-cymene are quite volatile by nature, so they would evaporate faster from the subject’s hand, leading to a loss of repellency after some time. The repellent activity of the *C. viminalis* EO against mosquitos can be deduced from work where the major compounds of *C. viminalis* were studied for their repellency against different mosquito species. For example, Klocke, et al. [30] reported that 1,8-cineole was a mild mosquito-feeding repellent against *Ae. aegypti*. Other studies showed that α-pinene possessed moderate repellent activity against *An. gambiae* [31] and *Ae. albopictus* [32]. A thorough literature search showed that *C. viminalis* has not been investigated for its repellent activity against mosquitoes; however, there are some reports of the insecticidal activities of the *C. viminalis* EO or extracts against pest insects. Yadav, et al. [33] showed that isopropanol, methanol, and acetone extracts of *C. viminalis* dried leaves acted as larvicides, with an LC_50_ of 71–115 ppm against the early third instar larvae of *Ae. albopictus*. In another study, the EO of *C. viminalis* decreased the growth rate of *Tribolium confusum* [34]. Other work on the EO of *C. viminalis* showed insecticidal activity against aphids [35]. In addition, a study from South Africa reported the insecticidal activity (LC_50_ = 0.019 μL/cm^3^) of the *C. viminalis* EO against *Callosobruchus maculatus* [36]. 

The *H. odoratissimum* EO showed moderate repellence against the tested species of mosquitoes. Interestingly, more repellence was observed in the case of *Cx. quinquefasciatus* compared to *Ae. aegypti* and *An. gambiae*. In a previous study, Ocheng, et al. [37] reported that the main compounds in the *H. odoratissimum* EO were α-pinene (4.2%), δ-cadinene (7.0%), β-caryophyllene (12.6%), α-copaene (7.3%), α-humulene (14.1%), levomenol (7.3%), and palmitic acid (27.1%). The combination of these major compounds, along with other minor compounds, could be the reason for its excellent and moderate mosquito-repellent activity against *Cx. quinquefasciatus* and *Ae. aegypti*, respectively. To our knowledge, *H. odoratissimum* EO has never been studied for its mosquito-repellent or larvicidal activities. However, some reports have documented the antibacterial and antifungal effects of *H. odoratissimum* [37,38,39,40]. Ocheng et al. (2014) [40] reported the excellent anti-bacterial activity of *H. odoratissimum* hexane extract, whereas Ocheng et al. (2015) [37] described the moderate anti-bacterial activity of *H. odoratissimum* EO. 

In the current study, the *H. suaveolens* EO showed the lowest repellence against the tested species of mosquitoes. Some studies reported the repellency activity of leaves EOs against *Ae. albopictus* [41], *An. gambiae*, and *Cx. quinquefasciatus* [42] mosquitoes.

In the current investigation, the most abundant compounds in *L. camara* EO were *trans*-β-caryophyllene (16.7%), sabinene (16.5%), 1,8-cineole (13.1%), α-humulene (8.6%), and nerolidol (5.5%). A study from India described 16.4% caryophyllene, 10.7% 1,8-cineole, 8,2% α-humulene, and 7.4% germacerence D in the *L. camara* leaves EO [43]. Another study from Côte d’Ivoire reported the seasonal variations in EOs extracted from the leaves and flowers of *L camara* and described 24.4–37.9% *trans*-β-caryophyllene, 3.0–10.9% sabinene, and 10.1–20.1% α-humulene as the most abundant compounds [44]. In the present study, we showed that the *C. viminalis* EO was composed of 15.5% α-pinene, 14% limonene, 12.4% α-phellandrene, 11.5% *p*-cymene, and 8.4% spathulenol, while a previous study from Egypt, by El-Sabrout, et al. [45], reported 1,8-cineole (71.8%), α-pinene (11.5%), and terpinen-4-ol (3.2%) as the major compounds of *C. viminalis*. In a study from India, reported by Srivastava, et al. [46], 1,8-cineole (61.7%), and α-pinene (24.2%) were the major components of the *C. viminalis* essential oil. Mubarak and his colleagues found that 1,8-cineole (61.5%) and α-pinene (21.5%) were the major compounds of *C. viminalis* [47]. The study from Brazil [48] revealed that the major components of the *C. viminalis* EO were 1,8-cineole (62.0%), α-pinene (21.7%), and α-terpineol (3.2%). Here, we reported that the *S. terebinthifolia* EO comprised 15.5% α-pinene, 14% limonene, 12.4% α-phellandrene, 11.5% *p*-cymene, and 8.4% spathulenol, while in a previous study from Brazil, 3-carene (55%), α-pinene (15%), sylvestrene (10.6%), and germacrene D (2.5%) were the major compounds in the EO of *S. terebinthifolia* fruits [49]. A similar composition of EO was reported from Tanzania [50] (Table S1). Another study from Egypt described that the EO of *S. terebinthifolia* fruits contained 36.9% α-pinene, 32.8% α-phellandrene, 11.9% limonene, and 6.0% α-terpineol [51]. A recent study from Morocco by Belhoussaine et al. reported that the EO distilled from the leaves of *S. terebenthifolia* was comprised of 23.2% limonene, 14.3% spathulenol, 13.3% cis-β-ocimene, and 9.4% each of α-terpinene and γ-terpinene [52]. Most of these main constituents were present in the EOs used in our study and in Belhoussaine et al.’s study [52], but in different relative proportions, except for α-pinene, which was not reported in the Moroccan study. The differences in the chemical composition of the EOs in the present and previous studies might be due to changes in the growing conditions and other biotic and a biotic factors [29].

Oviposition deterrents are considered an important tool in controlling mosquitoes. The current study’s results revealed that the EO of *L. camara* proved to be an excellent oviposition deterrent against *Ae. aegypti*, *An. gambiae*, and *Cx. quinquefasciatus*, compared to other tested EOs. The *L. camara* EO exhibited the highest deterrent effect against *Cx. quinquefasciatus* and the lowest effect against *An. gambiae*. The difference in oviposition deterrence towards different species of mosquitoes could be explained based on their chemoreceptor’s responses. Previously, the oviposition deterrent activity of *L. camara* has been observed against *An. gambiae*, where the EO of *L. camara* showed 98% oviposition deterrence when the egg laying media was treated with a 1 mL of 1000 ppm (1000 µg/mL) EO solution [53]. In the current study, however, 59% oviposition deterrence was observed against *An. gambiae* when a 300 µg/cm^2^ concentration was employed. The difference in the oviposition deterrent activity reported in the current and previous studies might be due to differences in the chemical compounds of EOs, differences in conducting the bioassay, as well as the tested concentration. It is well-documented that concentration plays an important role in determining the bioactivity of any substance. The EOs of *S. terebinthifolia*, *C. viminalis*, and *H. odoratissimum* also showed promising oviposition deterrence against *Ae. aegypti*, *An. gambiae*, and *Cx. quinquefasciatus*. Interestingly, all these EOs significantly affected the oviposition behaviour of *Cx. quinquefasciatus*, compared to other mosquito species, whereas *An. gambiae* was found to be less affected by the presence of these EOs. We know of no previous study on the oviposition deterrence effect of *S. terebinthifolia*, *C. viminalis*, and *H. odoratissimum.* However, there are studies where the major compounds of these EOs were tested for oviposition deterrence against some mosquito species. For example, limonene, β-pinene, and 1,8-cineole are proven oviposition deterrents of *Cx. quinquefasciatus*, *Spodoptera litura*, and *Chilo partellus* [54,55]. Female mosquitoes select the most suitable oviposition sites for the best fitness of their offspring [56,57]. The antennae of mosquitoes are replete with chemoreceptors that enable the insect to detect air-borne stimuli and assist in locating suitable sites for oviposition [58]. This is why female mosquitoes might prefer control cups compared to EOs-treated cups for oviposition. The results of our oviposition deterrence experiments showed that the tested EOs can prevent gravid mosquito females of *Ae. aegypti*, *An. gambiae*, and *Cx. quinquefasciatus* species from laying eggs in treated water.

## 4. Materials and Methods

### 4.1. Collection of Plant Material

Selected parts of *L. camara*, *S. terebinthifolia*, *C. viminalis*, and *H. suaveolens* were collected from natural populations in different areas of Pakistan. The aerial parts of *H. odoratissimum* were collected from Masaka, Uganda, during the flowering season. The details of plant material collection are presented in Table 2. The identity of the plants was authenticated by Dr. Abdul Nazir, a plant taxonomist at the Department of Environmental Sciences, COMSATS University Islamabad, Abbottabad Campus, Abbottabad, Pakistan. The plant material was shade-dried and stored at room temperature until used for essential oil extraction.

### 4.2. Extraction of Essential Oils

The steam distillation method was used to extract EOs from the collected plant material by using a Clevenger-type apparatus, as previously described [24]. A stainless-steel vessel was loaded with plant material (400 g) and two litres of distilled water was added to the bottom of the vessel to prevent direct contact of the plant material with water. The distillation vessel was heated by using an electric hotplate. Volatile compounds released from the plant material along with steam were cooled using a condenser fitted on the head of the vessel and the distillate was collected in a separating funnel for 3 h. The essential oil above the water layer was decanted and dried over anhydrous MgSO_4_. The extracted EO was weighed, and the percentage yield was calculated using the dry mass of the plant.

### 4.3. Rearing of Mosquitoes

Cultures of *Ae. aegypti*, *An. gambiae* s. l., and *Cx. quinquefasciatus* were reared in the laboratory using methods described in previous studies [12,24,59,60,61]. The larval population of mosquitoes was collected from the Health Department of Multan, Pakistan. Larvae were placed in a plastic container (20 × 16 × 4 cm) filled with 1 L water and fed with fish food (Osaka green fish food, India) containing 3% crude fat, 4% crude fibre, and 28% crude protein. Pupae were collected daily from the larval container and transferred to plastic cups containing 200 mL of tap water. The plastic cups were placed in Plexiglass cages (30 × 30 × 30 cm) for the emergence of adults. Cotton soaked with a 10% sucrose solution was placed in cages as an adult diet. After 4–5 days, females (*Ae. aegypti* and *Cx. quinquefasciatus*) were fed blood from a constrained pigeon placed in the adult cage while *An. gambiae* were fed human arm blood. Wax paper was placed on the inner side of the plastic jar filled with tap water, which was kept in the adult cage for oviposition. After oviposition, the immersed wax paper with eggs was transferred to the larval container, with 1000 mL of tap water for hatching. The rearing process continued until enough adults were obtained for the repellence and oviposition bioassays. The rearing of mosquito species was carried out in separate rooms. All rearing was carried out in a controlled room maintained at 25 ± 2 °C for *Ae. aegypti* and *Cx. quinquefasciatus*, while for *An. gambiae*, the room was maintained at 30 ± 3 °C. The relative humidity in the rearing room was maintained 80 ± 10%, with a photoperiod of 12 h:12 h light/dark.

### 4.4. Mosquito Repellency Bioassay

A human bait technique was used to test the repellence potential of EOs using a 1% *w*/*v* solution (33.3 µg/cm^2^ dose) and a 10% *w*/*v* solution (333 µg/cm^2^ dose) against *Ae. aegypti*, *An. gambiae* s. l., and *Cx. quinquefasciatus* females [24]. Solutions of each EO and DEET (99% purity, Sigma-Aldrich, St. Louis, MO, USA) were prepared using ethanol as a solvent. The DEET solution was used as a positive control and ethanol was used as a negative control. Twenty mated and blood-starved 4–5 days old female *Ae. aegypti* were released from the laboratory-reared colony in the experimental cage (30 × 30 × 30 cm) about 12 h before the start of the repellency bioassay. The hands of the subject (volunteer) were washed with scent-free liquid soap and allowed to dry for about 10 min before starting the bioassay. Plastic gloves were used to cover the subject’s hand except for the 30 cm^2^ circular area on the dorsal side of the hand. A 100 μL aliquot of the above-mentioned solution of the test substance or pure solvent as a negative control was evenly applied on the exposed area of the hand and dried in air for three min before exposing the hand to female *Ae. aegypti*. The subject’s hand was exposed to the female mosquitoes in the experimental cage, and mosquito landings were counted for 5 min. The experiment was repeated randomly five times for both the test samples and the negative control. The same procedure was followed to evaluate the repellency of EOs against *An. gambiae* and *Cx. quinquefasciatus*. The human volunteers were informed about the test procedure and consent was obtained before conducting repellence bioassays. The repellency percentage was calculated using the formula: percentage repellency = [(Mc − Mt)/Mc] × 100, where Mc is the number of mosquito landings on the negative control (solvent)-treated hand and Mt is the number of mosquito landings on the test substance-treated hand. The 10th generation of Asian strains of all three mosquito species were employed in this study. 

### 4.5. Time-Span Bioassays

Plant EOs that showed at least 50% repellence were further investigated to determine their repellent longevity against all three mosquito species. Time-span repellent bioassays were performed by following the same protocol as mentioned above in the repellency bioassay, except for the exposure of the same treated hand to the females of *Ae. aegypti*, *An. gambiae* s. l., or *Cx. quinquefasciatus* for 5 min after each 15 min time interval until the number of landings on control and treatment did not differ significantly. Time-span bioassays were conducted by using test samples at the dosages of 33.3 μg/cm^2^, and 333 μg/cm^2^. The experiments were repeated five times, and fresh female mosquitoes were employed for each replicate. A repellency bioassay for each mosquito species was conducted in separate climate-controlled rooms.

### 4.6. Oviposition Deterrence

The oviposition deterrence bioassay was conducted by adopting a method described by Soonwera [62]. Briefly, sixty 5–7 days old and blood-fed female mosquitoes were placed in a bioassay cage. Two plastic cups filled with 100 mL of distilled water were placed diagonally in the corners of the bioassay cage. One cup (250 mL) served as a test treatment while the other as a control. An aliquot of 300 µL of 10% ethanolic solution of an EO (*w*/*v*) was evenly sprayed on one side the wax paper strip (10 × 20 cm), air dried for 2 min, and then wrapped along the inner walls of the water-filled plastic cup, in such a way that the EO-treated area (10 × 10 cm) remained above the water level. The overall concentration of an EO on the treated wax paper was 300 µg/cm^2^. In the control cup, the solvent-treated filter paper was wrapped in the same way described for the test cup. After the application of a sample or solvent, the cups were left outside the cages so that the solvent could evaporate before the commencement of the experiment. The control and sample-treated cups were left in the adult mosquito cage for 48 h for oviposition. Afterwards, the eggs laid were counted in each cup. The position of the control and test cups was changed randomly to avoid the position effects on oviposition. We conducted oviposition tests five times by using a fresh mosquito population in each experiment.

### 4.7. Chemical Analysis of EOs

EOs that showed about 60% or higher repellency against all tested species of mosquitoes were analysed using a Hewlett Packard gas chromatograph connected to a mass spectrometer (GC-MS). The GC was equipped with a 30 m capillary column (DB-5, Agilent Technologies Inc., Santa Clara, CA, USA) with a 0.25 mm internal diameter and a stationary phase film thickness of 0.25 µm. The GC injector temperature was maintained isothermally at 235 °C throughout the sample analysis. The GC oven temperature was programmed as follows: an initial temperature of 40 °C set for 2 min, then increased to 240 °C at a rate of 4 °C per min, and finally maintained at 240 °C for 8 min. Helium was used as the mobile phase, at a constant flow rate of 1 mL/min through the column. An aliquot of 1 µL of dilute essential oil solution was injected into the GC, and the injector was operated in a splitless mode for 30 s. The MS was operated using the following parameters: an electron energy of 70 eV for ionization, an ion source temperature of 180 °C, and a mass spectrum scan range of 30–400 *m*/*z*. The total ion chromatogram was used to calculate the percent composition of compounds in EOs. A solution of a series of straight-chain alkanes (C_9_–C_24_) was injected in the GC-MS using the same parameters as used for the EOs analyses. The retention times of unknown compounds and alkanes were used to calculate the retention indices of separated compounds. The mass spectra and retention indices of separated compounds were initially compared to those available in the NIST-2008 MS library and webbook NIST online library to identify the separated compounds. Finally, identifications were verified by injecting the available pure standard compounds purchased from Sigma-Aldrich (St. Louis, MO, USA) and Alfa Aesar (Haverhill, MA, USA).

### 4.8. Statistical Analysis

A one-way ANOVA was used to evaluate statistical differences in the repellence and oviposition deterrent activity of the various EOs, followed by Tukey tests, at a significant threshold of alpha = 0.05 for pairwise comparisons of group means. Statistical analyses were performed using Statistica 8.1 software version 14.0.1.25 (TIBCO Software Inc., Palo Alto, CA, USA).

## 5. Conclusions

The EOs distilled from *L. camara* and *S. terebinthifolia* leaves displayed prolonged repellent activity, as well as greater oviposition deterrent activities against adult females of *Ae. aegypti*, *An. gambiae*, and *Cx. quinquefasciatus*. *Cx. quinquefasciatus* was found to be the most susceptible to repellency, as compared to *Ae. aegypti* and *An. gambiae.* In conclusion, the *L. camara* and *S. terebinthifolia* EOs have the potential to be natural mosquito repellents.

## Figures and Tables

**Table 1:** Percentage yield of EOs.

Scientific Name	Family	Location Coordinates	Part Used	Yield (%)
*Lantana camara* L.	Verbenaceae	30°20′3.6″ N 71°56′24″ E	Leaves	0.19
*Schinus terebinthifolia* Raddi.	Anacardiaceae	30°14′24″ N 71°26′24″ E	Leaves	0.09
*Callistemon viminalis* (Sol. ex Gaertn.) G.Don	Myrtaceae	30°20′24″ N 71°31′48″ E	Leaves, stems	0.21
*Helichrysum odoratissimum* L.	Asteraceae	0°19′32.8″ S 31°47′01.0″ E	Aerial parts	0.29
*Hyptis suaveolens* (L.) Poit.	Lamiaceae	30°19′12″ N 70°58′4.8″ E	Leaves	0.27

**Table 2:** Chemical composition of EOs based on the total ion chromatogram of GC-MS.

Retention Index	Compounds	*L. camara*	*S. terebinthifolia*	*C. viminalis*
922	α-Thujene	1.1	0.6	2.8
928	α-Pinene	4.3	15.5	34.2
942	Camphene	1.9	0.3	0.1
969	Sabinene	16.5	0.3	
971	β-Pinene	1.9	1.2	1.2
988	β-Myrcene	1.9	1	0.1
1001	α-Phellandrene	0.2	12.4	2.9
1007	3-Carene	2.4		0.2
1013	α-Terpinene	0.6	0.7	0.1
1021	*p*-Cymene	1.8	11.5	9
1026	Limonene	3.2	14	2.3
1027	1,8-Cineole	13.1		38.1
1036	*cis*-β-Ocimene	0.2		0.1
1056	γ-Terpinene	1	0.5	1.2
1064	*cis*-Sabinenhydrate	0.8		
1086	α-Terpinolene	0.3	0.8	0.5
1095	*trans*-Sabinene hydrate	0.2		
1099	Linalool	0.1	0.1	0.3
1103	2-Methylbutyl 2-methylbutanoate	0.2	0.1	
1135	*trans*-Pinocarveol	Tr	0.2	0.1
1140	Camphor	1	0.1	
1163	Borneol	0.7		
1175	4-Terpineol	1.1	0.4	0.5
1183	Cryptone	Tr	0.6	
1188	α-Terpineol	0.6		4
1199	Sabinol		1.1	
1205	Verbenone		0.3	
1218	*trans*-Carveol		0.2	
1250	*cis*-Ascaridol		0.2	
1272	Phellandral		0.2	
1302	Carvacrol		0.6	
1337	δ-Elemene	0.1	0.5	
1349	α-Cubebene	0.2	0.1	
1375	α-Copaene	0.4	0.2	
1384	β-Bourbonene	0	0.5	
1389	β-Cubebene	0.4		
1391	β-Elemene	0.2	4.5	
1419	*trans*-β-Caryophyllene	16.7	2	0.3
1428	β-Gurjunene	0.4	0.2	
1433	γ-Elemene	0.2	2.8	
1438	Aromadendrene	0	0.2	0.1
1443	γ-Gurjunene	0	0.1	
1453	α-Humulene	8.6	0.3	0.1
1460	Alloaromadendrene		0.3	0.1
1476	γ-Muurolene	0.3		
1480	Germacrene D	0.4	3.4	
1485	β-Selinene	0	0.6	
1496	Elixene	1	5.1	
1500	α-Muurolene	0.2		
1504	α-Selinene		0.2	
1523	δ-Cadinene	0.2	0.6	
1557	Davanone	1.2	2.8	
1564	*trans*-Nerolidol	5.5		
1577	Spathulenol	1.6	8.4	
1577	Ledene alcohol	0		0.7
1586	Caryophyllene oxide	0.5		
1629	Alloaromadendrene oxide	4.7	0.4	
1638	β-Cedren-9-α-ol		0.4	
1645	Nordracorhodin			0.1
1655	τ-Cadinol		0.4	
1658	Longifolenaldehyde	0.5		
1672	Aromadendrene oxide	0.4		
1675	Ledene oxide	0.2		
1704	Santalol	0.1		
1708	Cedren-13-ol		0.1	
1719	Isoaromadendrene epoxide		0.3	
1754	Globulol	0.1		
	Total Identified %	99.2	97.3	99.1

The retention index (RI) was determined using a DB-5 GC column.

## Data Availability

The original contributions presented in this study are included in the article. Further inquiries can be directed to the corresponding author.

## Supplementary Materials

The following supporting information can be downloaded at: https://www.mdpi.com/article/10.3390/molecules29112657/s1, Table S1: Studies reporting bioactivities of selected plants against different mosquito species.

## References

[1] Benelli G., Duggan M.F. (2018). Management of arthropod vector data–Social and ecological dynamics facing the One Health perspective. Acta Trop..

[2] Waggoner J.J., Gresh L., Vargas M.J., Ballesteros G., Tellez Y., Soda K.J., Sahoo M.K., Nuñez A., Balmaseda A., Harris E. (2016). Viremia and clinical presentation in Nicaraguan patients infected with Zika virus, chikungunya virus, and dengue virus. Clin. Infect. Dis..

[3] Kesete Y., Mhretab S., Tesfay M. (2020). Prevalence of malaria from blood smears examination: A three-year retrospective study from Nakfa Hospital, Eritrea. medRxiv.

[4] Sutthanont N., Attrapadung S., Nuchprayoon S. (2019). Larvicidal activity of synthesized silver nanoparticles from *Curcuma zedoaria* essential oil against *Culex quinquefasciatus*. Insects.

[5] Rani L., Thapa K., Kanojia N., Sharma N., Singh S., Grewal A.S., Srivastav A.L., Kaushal J. (2021). An extensive review on the consequences of chemical pesticides on human health and environment. J. Clean. Produc..

[6] Andreazza F., Oliveira E.E., Martins G.F. (2021). Implications of sublethal insecticide exposure and the development of resistance on mosquito physiology, behavior, and pathogen transmission. Insects.

[7] Afify A., Potter C. (2020). Insect repellents mediate species-specific olfactory behaviours in mosquitoes. Malar. J..

[8] Qiu H., Jun H.W., Dzimianski M., McCall J. (1997). Reduced transdermal absorption of N, N-diethyl-m-toluamide from a new topical insect repellent formulation. Pharm. Dev. Technol..

[9] Calafat A.M., Baker S.E., Wong L.-Y., Bishop A.M., Morales-A P., Valentin-Blasini L. (2016). Novel exposure biomarkers of N, N-diethyl-m-toluamide (DEET): Data from the 2007–2010 National Health and Nutrition Examination Survey. Environ. Int..

[10] Oftadeh M., Sendi J.J., Ebadollahi A., Setzer W.N., Krutmuang P. (2021). Mulberry protection through flowering-stage essential oil of *Artemisia annua* against the lesser mulberry pyralid, *Glyphodes pyloalis* Walker. Foods.

[11] Manh H.D., Tuyet O.T. (2020). Larvicidal and repellent activity of *Mentha arvensis* L. essential oil against *Aedes aegypti*. Insects.

[12] Abbas M.G., Haris A., Binyameen M., Nazir A., Mozūratis R., Azeem M. (2023). Chemical Composition, Larvicidal and Repellent Activities of Wild Plant Essential Oils against *Aedes Aegypti*. Biology.

[13] Sharma R., Rao R., Kumar S., Mahant S., Khatkar S. (2019). Therapeutic potential of citronella essential oil: A review. Curr. Drug Discov. Technol..

[14] Carroll S.P., Loye J. (2006). PMD, a registered botanical mosquito repellent with deet-like efficacy. J. Am. Mosq. Control Assoc..

[15] Tripathi A.K., Upadhyay S., Bhuiyan M., Bhattacharya P.R. (2009). A review on prospects of essential oils as biopesticide in insect-pest management. J. Pharmacogn. Phytother..

[16] Amer A., Mehlhorn H. (2006). Repellency effect of forty-one essential oils against *Aedes*, *Anopheles*, and *Culex* mosquitoes. Parasitol. Res..

[17] Govindarajan M., Sivakumar R., Rajeswari M., Yogalakshmi K. (2012). Chemical composition and larvicidal activity of essential oil from *Mentha spicata* (Linn.) against three mosquito species. Parasitol. Res..

[18] Negahban M., Moharramipour S., Sefidkon F. (2007). Fumigant toxicity of essential oil from *Artemisia sieberi* Besser against three stored-product insects. J. Stored Prod. Res..

[19] Kweka E.J., Munga S., Mahande A.M., Msangi S., Mazigo H.D., Adrias A.Q., Matias J.R. (2012). Protective efficacy of menthol propylene glycol carbonate compared to N, N-diethyl-methylbenzamide against mosquito bites in Northern Tanzania. Parasit. Vectors.

[20] Haris A., Azeem M., Abbas M.G., Mumtaz M., Mozūratis R., Binyameen M. (2023). Prolonged repellent activity of plant essential oils against dengue vector, Aedes aegypti. Molecules.

[21] Dua V., Gupta N., Pandey A., Sharma V. (1996). Repellency of *Lantana camara* (Verbenaceae) flowers against *Aedes* mosquitoes. J. Am. Mosq. Control Assoc..

[22] Keziah E.A., Nukenine E.N., Danga S.P.Y., Younoussa L., Esimone C.O. (2015). Creams formulated with *Ocimum gratissimum* L. and *Lantana camara* L. crude extracts and fractions as mosquito repellents against *Aedes aegypti* L.(Diptera: Culicidae). J. Insect Sci..

[23] Bhargava S., Agrawal D., Agrawal O. (2013). Repellent activity of essential oil and leaf extract of *Lantana camara* L. in laboratory condition. Int. J. Theor. Appl. Sci..

[24] Azeem M., Zaman T., Tahir M., Haris A., Iqbal Z., Binyameen M., Nazir A., Shad S.A., Majeed S., Mozūraitis R. (2019). Chemical composition and repellent activity of native plants essential oils against dengue mosquito. Aedes Aegypti. Ind. Crops Prod..

[25] Iqbal S., Khan F.A., Haris A., Mozūratis R., Binyameen M., Azeem M. (2023). Essential oils of four wild plants inhibit the blood seeking behaviour of female *Aedes aegytpi*. Exp. Parasitol..

[26] Rao J., Chen B., McClements D.J. (2019). Improving the efficacy of essential oils as antimicrobials in foods: Mechanisms of action. Ann. Rev. Food Sci. Technol..

[27] Laznik Z., Vidrih M., Trdan S. (2012). Efficacy of four essential oils against *Sitophilus granarius* (L.) adults after short-term exposure. Afr. J. Agric. Res..

[28] Nenaah G.E., Almadiy A.A., Al-Assiuty B.A., Mahnashi M.H. (2022). The essential oil of Schinus terebinthifolius and its nanoemulsion and isolated monoterpenes: Investigation of their activity against *Culex pipiens* with insights into the adverse effects on non-target organisms. Pest Manag. Sci..

[29] Parveen A., Abbas M.G., Keefover-Ring K., Binyameen M., Mozūraitis R., Azeem M. (2024). Chemical Composition of Essential Oils from Natural Populations of Artemisia scoparia Collected at Different Altitudes: Antibacterial, Mosquito Repellent, and Larvicidal Effects. Molecules.

[30] Klocke J.A., Darlington M.V., Balandrin M.F. (1987). 1, 8-Cineole (Eucalyptol), a mosquito feeding and ovipositional repellent from volatile oil of *Hemizonia fitchii* (Asteraceae). J. Chem. Ecol..

[31] Okoli B.J., Ladan Z., Mtunzi F., Hosea Y.C. (2021). *Vitex negundo* L. Essential oil: Odorant binding protein efficiency using molecular docking approach and studies of the mosquito repellent. Insects.

[32] Giatropoulos A., Papachristos D.P., Kimbaris A., Koliopoulos G., Polissiou M.G., Emmanouel N., Michaelakis A. (2012). Evaluation of bioefficacy of three Citrus essential oils against the dengue vector *Aedes albopictus* (Diptera: Culicidae) in correlation to their components enantiomeric distribution. Parasitol. Res..

[33] Yadav R., Tyagi V., Tikar S.N., Sharma A.K., Mendki M.J., Jain A.K., Sukumaran D. (2014). Differential larval toxicity and oviposition altering activity of some indigenous plant extracts against dengue and chikungunya vector *Aedes albopictus*. Entomol. Res..

[34] Hamzavi F., Moharramipour S. (2017). Chemical composition and antifeedant activity of essential oils from Eucalyptus camaldulensis and Callistemon viminalis on *Tribolium confusum*. Int. J. Agric. Technol..

[35] Sales T.A., Cardoso M.D.G., Guimarães L.G.D.L., Camargo K.C., Rezende D.A., Brandão R.M., Souza R.V., Ferreira V.R., Marques A.E., Magalhães M.L. (2017). Essential oils from the leaves and flowers of *Callistemon viminalis*: Chemical characterization and evaluation of the insecticide and antifungal activities. Am. J. Plant Sci..

[36] Ndomo A., Tapondjou L.A., Ngamo L., Hance T. (2010). Insecticidal activities of essential oil of *Callistemon viminalis* applied as fumigant and powder against two bruchids. J. Appl. Entomol..

[37] Ocheng F., Bwanga F., Joloba M., Softrata A., Azeem M., Pütsep K., Borg-Karlson A.-K., Obua C., Gustafsson A. (2015). Essential oils from ugandan aromatic medicinal plants: Chemical composition and growth inhibitory effects on oral pathogens. Evid. Based Complement. Altern. Med..

[38] Lawal O.A., Ogunwande I.A., Kasali A.A., Opoku A.R., Oyedeji A.O. (2015). Chemical composition, antibacterial and cytotoxic activities of essential oil from the leaves of *Helichrysum odoratissimum* grown in South Africa. J. Essent. Oil Bear. Plants.

[39] Zantanta N., Kambizi L., Etsassala N.G., Nchu F. (2022). Comparing crop yield, secondary metabolite contents, and antifungal activity of extracts of *Helichrysum odoratissimum* cultivated in aquaponic, hydroponic, and field systems. Plants.

[40] Ocheng F., Bwanga F., Joloba M., Borg-Karlson A.-K., Gustafsson A., Obua C. (2014). Antibacterial activities of extracts from Ugandan medicinal plants used for oral care. J. Ethnopharmacol..

[41] Benelli G., Flamini G., Fiore G., Cioni P.L., Conti B. (2013). Larvicidal and repellent activity of the essential oil of *Coriandrum sativum* L. (Apiaceae) fruits against the filariasis vector *Aedes albopictus* Skuse (Diptera: Culicidae). Parasitol. Res..

[42] Abagli A.Z., Alavo T.B.C. (2011). Essential oil from bush mint, *Hyptis suaveolens*, is as effective as DEET for personal protection against mosquito bites. Open Entomol. J..

[43] Dua V., Pandey A., Dash A. (2010). Adulticidal activity of essential oil of *Lantana camara* leaves against mosquitoes. Indian J. Med. Res..

[44] Nea F., Kambiré D.A., Genva M., Tanoh E.A., Wognin E.L., Martin H., Brostaux Y., Tomi F., Lognay G.C., Tonzibo Z.F. (2020). Composition, seasonal variation, and biological activities of *Lantana camara* essential oils from Côte d’Ivoire. Molecules.

[45] El-Sabrout A.M., Zoghroban A.A., Abdelgaleil S.A. (2020). Chemical composition and effects of four essential oils on mortality, development and physiology of the West Nile virus vector, *Culex pipiens*. Int. J. Trop. Insect Sci..

[46] Srivastava S., Ahmad A., Syamsunder K., Aggarwal K., Khanuja S. (2003). Essential oil composition of *Callistemon viminalis* leaves from India. Flavour Fragr. J..

[47] Mubarak E.E., Mohajer S., Ahmed I., Taha R.M. (2014). Essential oil compositions from leaves of *Eucalyptus camaldulensis* Dehn. and *Callistemon viminalis* Originated from Malaysia. IPCBEE.

[48] Oliveira J.A.D., Garcia I.P., Corrêa E.J.A., de Lima L.H.F., Santos H.D., de Assis R.M.A., Pinto J., Bertolucci S.K.V. (2023). Larvicidal susceptibility of essential oils from *Cinnamodendron dinisii*, *Callistemon viminalis* and *Myrcia tomentosa* against Culex quinquefasciatus (Say) (Diptera: Culicidae). South Afr. J. Bot..

[49] Silva A.G., Almeida D.L., Ronchi S.N., Bento A.C., Scherer R., Ramos A.C., Cruz Z.M. (2010). The essential oil of Brazilian pepper, *Schinus terebinthifolia* Raddi in larval control of *Stegomyia aegypti* (Linnaeus, 1762). Parasit. Vectors.

[50] Kweka E.J., Nyindo M., Mosha F., Silva A.G. (2011). Insecticidal activity of the essential oil from fruits and seeds of *Schinus terebinthifolia* Raddi against African malaria vectors. Parasit. Vectors.

[51] Hussein H.S., Salem M.Z., Soliman A.M. (2017). Repellent, attractive, and insecticidal effects of essential oils from *Schinus terebinthifolius* fruits and *Corymbia citriodora* leaves on two whitefly species, *Bemisia tabaci*, and *Trialeurodes ricini*. Sci. Hortic..

[52] Belhoussaine O., El Kourchi C., Harhar H., Bouyahya A., El Yadini A., Fozia F., Alotaibi A., Ullah R., Tabyaoui M. (2022). Chemical composition, antioxidant, insecticidal activity, and comparative analysis of essential oils of leaves and fruits of *Schinus molle* and *Schinus terebinthifolius*. Evid. Based Complement. Altern. Med..

[53] Wangrawa D.W., Badolo A., Guelbeogo W.M., Nebie R.C.H., Sagnon N.F., Borovsky D., Sanon A. (2021). Larvicidal, oviposition-deterrence, and excito-repellency activities of four essential oils: An eco-friendly tool against malaria vectors *Anopheles coluzzii* and *Anopheles gambiae* (Diptera: Culicidae). Int. J. Trop. Insect Sci..

[54] Koul O., Singh R., Kaur B., Kanda D. (2013). Comparative study on the behavioral response and acute toxicity of some essential oil compounds and their binary mixtures to larvae of *Helicoverpa armigera*, *Spodoptera litura* and *Chilo partellus*. Ind. Crops Prod..

[55] Andrade-Ochoa S., Sánchez-Aldana D., Chacón-Vargas K.F., Rivera-Chavira B.E., Sánchez-Torres L.E., Camacho A.D., Nogueda-Torres B., Nevárez-Moorillón G.V. (2018). Oviposition deterrent and larvicidal and pupaecidal activity of seven essential oils and their major components against *Culex quinquefasciatus* Say (Diptera: Culicidae): Synergism–antagonism effects. Insects.

[56] Soonwera M., Phasomkusolsil S. (2017). Adulticidal, larvicidal, pupicidal and oviposition deterrent activities of essential oil from *Zanthoxylum limonella* Alston (Rutaceae) against *Aedes aegypti* (L.) and *Culex quinquefasciatus* (Say). Asian Pac. J. Trop. Biomed..

[57] Hwang Y.-S., Kramer W.L., Mulla M.S. (1980). Oviposition attractants and repellents of mosquitoes: Isolation and identification of oviposition repellents for *Culex* mosquitoes. J. Chem. Ecol..

[58] Davis E.E. (1976). A receptor sensitive to oviposition site attractants on the antennae of the mosquito, *Aedes aegypti*. J. Insect Physiol..

[59] Bhujel P., Saha D. (2023). Rearing Protocol for *Culex quinquefasciatus*. Sci. Vis..

[60] Zheng M.-L., Zhang D.-J., Damiens D.D., Lees R.S., Gilles J.R. (2015). Standard operating procedures for standardized mass rearing of the dengue and chikungunya vectors *Aedes aegypti* and *Aedes albopictus* (Diptera: Culicidae)-II-Egg storage and hatching. Parasit. Vectors.

[61] Govindarajan M., Sivakumar R., Rajeswary M., Yogalakshmi K. (2013). Chemical composition and larvicidal activity of essential oil from *Ocimum basilicum* (L.) against *Culex tritaeniorhynchus*, *Aedes albopictus* and *Anopheles subpictus* (Diptera: Culicidae). Exp. Parasitol..

[62] Soonwera M. (2015). Larvicidal and oviposition deterrent activities of essential oils against house fly (*Musca domestica* L.; Diptera: Muscidae). J. Agric. Technol..

