# Supplementary material for: Chemical Composition, Repellent, and Oviposition Deterrent Potential of Wild Plant Essential Oils against Three Mosquito Species
Source: Molecules. 2024 Jun 4;29(11):2657. doi: 10.3390/molecules29112657 (PMC11173646; doi:10.3390/molecules29112657)
Supplement: Supplementary file 1 [file molecules-29-02657-s001.zip › molecules-2997239-supplementary.pdf]

Table S1. Studies reporting bioactivities of selected plants against different mosquito species

Plants	Country	Essential oil/ solvent extract	Mosquito	Concentration/Dose Tested	Bioactivity (efficacy %)	Major chemical components	Reference
Lantna camara	India	Leaves essential oil	*Aedes aegypti*	100 μg/cm²	Adulticidal (93)	caryophyllene (16.4%), eucalyptol (10.7%), α-humelene (8.2%), germacrene (7.4%)	[1]
			Culex Quinquefasciatus		Adulticidal (95)		
			Anopheles Culicifacies		Adulticidal (100)		
			An. Fluviatilis		Adulticidal (100)		
			An. Stephensi		Adulticidal (100)		
		Leaves essential oil	*Ae. aegypti*	1660 μg/cm²	Repellence (100)	Not reported	[2]
		Leaves alcoholic extract					
		Leave ether extract					
		Leaves hexane extract					

	Nigeria	Leaves methanol extract		8000 μg/cm²	Repellence (100%)	Not reported	[3]
		Leaves Ethyl acetate fraction					
		Leaves hexane fraction					
Schinus terebinthifolia	Tanzania	Fruit essential oil	*An. gambiae*	2625 μg/mL	Larvicidal (97)	3-carene (55.4%), α-pinene (15.6%), sylvestrene (10.7%), germacrene D (2.5%)	[4]
			Cx. quinquefasciatus		Larvicidal (96)		
	Brazil	Fruit essential oil	*Stegomyia aegypti*	862 μg/mL	Larvicidal (100)	3-carene (55.4%), α-pinene (16.3%), sylvestrene (10.7%), germacrene D (2.2%), β-myrcene	[5]
	Saudi Arabia	Fruit essential oil	*Cx. pipiens*	60 μL/L	Larvicidal (100)	3-carene (31.2%), α-pinene (15.3%), α-phellandrene	[6]
				0.5 μL/cm²	Repellence (100)		

				30 μL/L	Adulticidal (100)	(11.2%), limonene (9.3%)	
Callistemon viminalis	India	Leave isopropanol extract	*Ae. albopictus*	71.3 ppm	Larvicidal (50)	Not Reported	[7]
		Leave acetone extract		110 ppm			
		Leave methanol extract		115 ppm			
	Egypt	Leaves essential oil	*Cx. pipiens*	100 mg/L	Larvicidal (10.7)	1,8-cineol (71.8%), α-pinene (11.5%), α-terpineol (3.2%)	[8]
					Adulticidal (11.7)		
	Brazil	Leaves essential oil	*Cx. quinquefasciatus*	398.6 μg/mL	Larvicidal (50)	1,8-cineol (62.0%), α-pinene (21.7%), α-terpineol (3.2%)	[9]
Hyptis suaveolens	Italy	Leaves essential oil	*Ae. albopictus*	450 ppm	Larvicidal (98)	sabinene (21.9%), β-caryophyllene (16.1%), terpinolene (9.6%), 4-terpineol (7.3%)	[10]
				0.75 μg/cm^2	Repellence (100)		

	Benin	Leaves essential oil	*An. gambiae*	10%	Repellence (100)	Not reported	[11]
			Cx. quinquefasciatus				

References

1. Dua, V.; Pandey, A.; Dash, A., Adulticidal activity of essential oil of *Lantana camara* leaves against mosquitoes. *Indian Journal of Medical Research* **2010,** 131, (3), 434-439.
2. Bhargava, S.; Agrawal, D.; Agrawal, O., Repellent activity of essential oil and leaf extract of *Lantana camara* L. in laboratory condition. *International Journal of Theoretical and Applied Science* **2013,** 5, (1), 170-174.
3. Keziah, E. A.; Nukenine, E. N.; Danga, S. P. Y.; Younoussa, L.; Esimone, C. O., Creams formulated with *Ocimum gratissimum* L. and *Lantana camara* L. crude extracts and fractions as mosquito repellents against *Aedes aegypti* L.(Diptera: Culicidae). *Journal of Insect Science* **2015,** 15, (1).
4. Kweka, E. J.; Nyindo, M.; Mosha, F.; Silva, A. G., Insecticidal activity of the essential oil from fruits and seeds of *Schinus terebinthifolia* Raddi against African malaria vectors. *Parasites & Vectors* **2011,** 4, (1), 1-10.
5. Silva, A. G.; Almeida, D. L.; Ronchi, S. N.; Bento, A. C.; Scherer, R.; Ramos, A. C.; Cruz, Z. M., The essential oil of Brazilian pepper, *Schinus terebinthifolia* Raddi in larval control of *Stegomyia aegypti* (Linnaeus, 1762). *Parasites & vectors* **2010,** 3, 1-7.
6. Nenaah, G. E.; Almadiy, A. A.; Al-Assiuty, B. A.; Mahnashi, M. H., The essential oil of Schinus terebinthifolius and its nanoemulsion and isolated monoterpenes: investigation of their activity against *Culex pipiens* with insights into the adverse effects on non-target organisms. *Pest Management Science* **2022,** 78, (3), 1035-1047.
7. Yadav, R.; Tyagi, V.; Tikar, S. N.; Sharma, A. K.; Mendki, M. J.; Jain, A. K.; Sukumaran, D., Differential larval toxicity and oviposition altering activity of some indigenous plant extracts against dengue and chikungunya vector *Aedes albopictus*. *Entomology Research* **2014,** 2, 212-220.
8. El-Sabrout, A. M.; Zoghroban, A. A.; Abdelgaleil, S. A., Chemical composition and effects of four essential oils on mortality, development and physiology of the West Nile virus vector, *Culex pipiens*. *International Journal of Tropical Insect Science* **2020,** 40, (4), 789-799.
9. Oliveira, J. A. d. C.; Garcia, I. P.; Corrêa, E. J. A.; de Lima, L. H. F.; Santos, H. d. L.; de Assis, R. M. A.; Pinto, J. E. B. P.; Bertolucci, S. K. V., Larvicidal susceptibility of essential oils from Cinnamodendron dinisii, Callistemon viminalis and Myrcia tomentosa against Culex quinquefasciatus (Say) (Diptera: Culicidae). *South African Journal of Botany* **2023,** 163, 95-104.
10. Conti, B.; Benelli, G.; Flamini, G.; Cioni, P. L.; Profeti, R.; Ceccarini, L.; Macchia, M.; Canale, A., Larvicidal and repellent activity of *Hyptis suaveolens* (Lamiaceae) essential oil against the mosquito *Aedes albopictus* Skuse (Diptera: Culicidae). *Parasitology research* **2012,** 110, 2013-2021.

11. Abagli, A.; Alavo, T., Essential oil from bush mint, *Hyptis suaveolens*, is as effective as DEET for personal protection against mosquito bites. *Open Entomol. J.* **2011,** 5, (1), 232-243.
